# Why we should move beyond treating the Impostor Phenomenon as categorical construct and instead adopt the continuous approach

**DOI:** 10.3389/fpsyg.2026.1991425

**Published:** 2026-10-15

**Authors:** Kay Brauer

**Affiliations:** Psychological Assessment and Differential Psychology, Department of Psychology, Martin Luther University Halle-Wittenberg, Halle, Germany

**Keywords:** categorical approach, CIPS, clance impostor phenomenon scale, distribution, impostor phenomenon, prevalence, cut-off

## Abstract

The Impostor Phenomenon (IP) is a psychological trait that receives increasing interest across study disciplines. Although the IP is conceptualized as an individual difference variable that describes expressions on a continuum from low to high expressions, some studies still treat the IP as categorical. One consequence of this approach is that prevalence rates are estimated based on arbitrary and unvalidated cutoffs. I discuss the theoretical and methodological flaws of this approach, which are also evident when considering findings from three recent meta-analyses of the IP. Further, I suggest adopting the continuous approach to the IP and illustrate, based on data from 137 samples (*N* = 22,552), that aims such as testing differences between populations (i.e., students and employees) and whether the IP increased over time can be well addressed using distributional parameters. I discuss implications of this approach for the field.

## 1 Introduction

The Impostor Phenomenon (IP; Clance, 1985) describes individual differences in experiences of intellectual phoniness among individuals who have access to evidence supporting their capabilities (e.g., feedback from colleagues and supervisors or grades). Accordingly, those with high levels of IP externalize their success (e.g., Brauer and Proyer, 2022) and fear being exposed as intellectual frauds when being assigned new tasks. Individual differences in the IP are described along a continuum from low to high levels of expression. The IP has received increasing interest (Stone-Sabali et al., 2023), and while the field is generally flourishing and advancing knowledge about the IP and its correlates and consequences, the practice of treating the IP as a categorical construct and the efforts to estimate “prevalence rates” do not align with current knowledge about the IP and have methodological flaws. I will illustrate these issues from theoretical and empirical perspectives, with a focus on meta-analyses, and demonstrate that the continuous approach should be favored based on data from 137 samples and 22,552 participants.

## 2 Conceptual issues

Clance and Imes (1978) introduced the IP based on observations from their work as psychotherapists. Although the IP has been first described from a clinical perspective, Pauline Clance has clarified that the IP is not a disorder but describes individual differences in experiences of how individuals perceive their abilities and experience anxiousness about being exposed as intellectual fraud (Clance and Lawry, 2024).^1^ Further, from a conceptual perspective there is consensus that the IP is *not* a clinical entity, as it is not included in classifications such as the *Diagnostic and Statistical Manual of Mental Disorders* (American Psychiatric Association, 2013) and the *International Classification of Diseases* (World Health Organization, 2022). Accordingly, the IP is not a diagnosis, and the frequently used description of someone or a portion of a population as “having the IP” contradicts the conceptualization of the IP in principle.^2^ Finally, considering the available evidence on typical “prevalence rates” of the IP (50 to 60%), it has been argued that this points to the conclusion that the IP is a normative experience that is better conceptualized as a trait variable than a categorical construct (Brauer and Proyer, 2026). Hence, theoretical accounts do not support the notion that the IP is a dichotomous or categorical construct.

## 3 Reliance on arbitrary and unvalidated cutoffs

Although there is no conceptual support for the notion that the IP should be viewed as categorical or clinical entity, numerous studies report “prevalence rates” (i.e., the portion of a population that is affected by a syndrome) when studying the IP. It can be assumed that this practice has mostly historic reasons because when Clance (1985) introduced the 20-item self-report questionnaire, the Clance Impostor Phenomenon Scale (CIPS), which remains the state-of-the-art measure of the IP to date, she also suggested cutoff scores. CIPS scores can vary between 20 and 100 points and a score of 62 has typically been used as a cutoff. This approach implies that people can be empirically categorized as “nonimpostors” and “impostors” on basis of CIPS scores. However, studies differ in how they set the cutoff and have relied on arbitrary thresholds, with some studies using 61 or 63 points, some use the empirical mean of a study sample, and others rely on the empirical median. Holmes et al. (1993) examined the 62-points-cutoff in relation to classifications after interviews but relied on a very small sample size of 62 participants, rendering the findings as lacking robustness and generalizability. Taking aside that the choice of these cutoffs is arbitrary, they lack empirical support, and recent findings have highlighted that they do not converge well (see Brauer and Proyer, 2026).^3^

One might argue that the inspection of CIPS score distributions could provide further insight into whether the individual difference assumption holds, or whether skewed or bimodal distributions support a categorical approach to the IP. Most studies report that the CIPS scores are normally distributed across samples (e.g., Brauer and Proyer, 2023; Brauer and Wolf, 2016; Jefferson et al., 2024). Accordingly, estimating “prevalence rates” of the IP is neither theoretically nor empirically justified, given the lack of theoretical support for a categorical conceptualization and the absence of validated thresholds that would allow researchers to reliably distinguish between individuals with low and high levels of IP.

## 4 Same same but different and the consistency of inconsistency

The consequences of relying on theoretically and empirically unjustified CIPS cutoffs can be demonstrated based on findings from three recently published meta-analyses of IP “prevalence rates.” Salari et al. (2025) studied the population of health service providers (*k* = 30 studies; *N* = 11,483) and Omar et al. (2026); *k* = 34 studies, *N* = 9,550 and Vera-Ponce et al. (2026); *k* = 30 studies, *N* = 9,550 analyzed data from medical students. Their approach is straight-forward, as they aggregate findings on the prevalence rates from the included studies and report about the frequency distribution, with most findings being based on the CIPS (e.g., 26 of 30 eligible studies in Vera-Ponce et al.’s study). The studies report “prevalence rates” of 54.3% (Vera-Ponce et al., 2026), 49% (Omar et al., 2026), and 64.5% (Salari et al., 2025). Hence, roughly about half of the participants are classified as showing higher IP expressions, which is what one would expect when placing a threshold near the mean of a normally distributed variable.

However, the interpretation of these prevalence rates requires considerable caution. Setting aside the theoretical concern that the IP is not conceptualized as categorical construct and the fact that the cutoffs were not validated, a major issue is that studies differ in how they define their cutoffs. As Vera-Ponce et al. (2026) note “The inclusion of studies using CIPS thresholds of ≥61, ≥ 62, or ≥ 63 introduces variability in defining threshold-defined IP.” (p: 15) While the acknowledgment of this issue should be applauded, their subsequent notion “[…]cut-off points (≥61 or ≥63) were treated as comparable because they fall within the same conventional CIPS severity range and do not represent meaningfully different categories on the 20-item scale” (p. 15) does not hold. Brauer and Proyer (2026) illustrated the consequences of relying on the arbitrary cut-offs and showed, based on two openly available data sets comprising 1,124 and 670 participants, respectively, that the choice of the cutoff robustly affects estimates. For example, when comparing the cutoffs of 60 points to those placed at 58 and 62 points, prevalence rates vary from 4.6 to 14.1% when applied to data from the *same* samples. Accordingly, the choice of cutoff strongly influences prevalence rates, particularly when comparing rates from *different* samples using *different* cutoffs, as was done in the recent meta-analyses of the IP.

The concern about the arbitrary nature of the cutoffs and the lack of consistency is supported by all three meta-analyses, showing high variability in point estimates. It can be assumed that the reliance on arbitrary cutoffs contributes to the high variability of the observed “prevalence rates.” The three meta-analyses report the heterogeneity index *I*^2^ (also informally referred to as the inconsistency index), which ranges from 0 to 100%. Higher values indicate higher variability across studies that cannot be explained by sampling error alone; to the point that confidence intervals between the studies included in the meta-analysis show poor overlap (Higgins and López-López, 2026). Vera-Ponce et al. (2026) report an *I*^2^ of 98%, which is consistent with the coefficients of 98.6 and 95.6% reported by Salari et al. (2025) and Omar et al. (2026), respectively. These values indicate substantial heterogeneity between the studies included in the respective meta-analyses and warrant considerable caution when interpreting the pooled point estimates. Considering that the populations studied in each meta-analysis (medical students and health service providers) are comparatively homogenous and given the demonstrated influence of cutoff selection on the estimates (see Brauer and Proyer, 2026), it is plausible that the inclusion of different arbitrary cutoffs to the consistently inconsistent findings across studies when estimating “prevalence rates” of the IP. Hence, even when setting aside the lack of conceptual support for treating the IP as categorical construct, the findings provide limited grounds for drawing conclusions about IP prevalence rates from a methodological perspective.

## 5 The field should refrain from dichotomizing the Impostor Phenomenon

Considering the theoretical flaws of treating the IP as categorical construct and the findings from three large meta-analyses highlighting the consistent inconsistency resulting from the use of arbitrary and unvalidated cutoffs for the standard IP measure, I argue that that the field should refrain from adopting a categorical approach. Instead, research should move forward by studying the IP in alignment with its theoretical and empirical conceptualization, namely, as an individual differences variable that describes IP experiences on a continuum from low to high levels of expression. I fully acknowledge that cutoffs can be useful for the study of individual differences variables, for example, when one is interested in analyzing group differences between low and high-scorers. However, unless cutoffs that have been validated by multimethodological approaches, I recommend that the field focuses on the continuous conceptualization of the IP, which is robustly supported by theory and data.

## 6 (Meta-)analyzing the Impostor Phenomenon with the continuous approach

Most studies investigating the IP use the continuous approach, and I suggest basing future meta-analyses on the sample means and *SD*s whenever possible. This has several merits. Aside from the typical restrictions that go along with artificially dichotomizing continuous data (e.g., loss of information and reduced statistical power; Altman and Royston, 2006; Dawson and Weiss, 2012; Royston et al., 2006; MacCallum et al., 2002), this approach does not depend on arbitrary and unvalidated cutoffs that cannot be readily compared across studies.

I provide an illustration of addressing two frequently discussed research questions using a meta-analytic approach based on continuous CIPS scores. First, it has been discussed whether students and working professionals differ in their levels of IP expressions (e.g., Brauer and Proyer, 2023; Neureiter and Traut-Mattausch, 2016). Accordingly, I will examine whether students and employees differ in their CIPS scores. Second, an open question is whether IP expressions have increased over time, with studies showing mixed findings when relying on “prevalence rates” to address this question (Salari et al., 2025; Vera-Ponce et al., 2026).

To illustrate this approach, I considered the studies of nonclinical samples identified in Stone-Sabali et al. (2023) bibliographic review of the IP literature published between 1978 and 2022. The inclusion criteria were that the samples (a) completed the 20-item standard instrument (CIPS), (b) reported sample sizes and sample means of the CIPS scores, and (c) could be unambiguously coded as students, employees, or mixed samples. This resulted in 75 student samples (*n* = 13,383), 50 employee samples (*n* = 5,521), and 12 mixed samples (*n* = 3,648). All analyses are based on the sample means and use sample sizes as weighting factor because larger samples provide more accurate estimates than smaller samples (Lipsey and Wilson, 2001). Further, I computed standard errors and confidence intervals on basis of bootstrapping, which estimates the uncertainty by repeatedly resampling the data from the data set. In the present analysis, 5,000 resamples were drawn. Note that this data set is used for illustration and does not substitute a classical meta-analysis.

A first analysis using all 137 samples, comprising 22,552 participants, showed that the mean of the samples’ CIPS score distributions was 59.3 points, with a 95% confidence interval (CI) of [59.3, 59.5] and an *SD* of 6.07. The distribution showed a skewness of −0.03 and kurtosis of 0.04. Hence, the distribution of mean CIPS scores across the 137 samples was approximatively normal, covering a range from 42.0 and 75.2 points. This finding further supports the notion that the IP is best understood on a continuum between low to high levels of expressions rather than a dichotomous phenomenon that people either “have” or do not “have,” when considering the sample level.

To address the question of whether students and employees differ in their IP expressions, I analyzed and compared the means of the students (*k* = 75 samples; *n* = 13,383) and employee samples (*k* = 50; *n* = 5,521). A first inspection of the descriptive statistics showed that students and professionals had comparable mean CIPS scores, with *M* = 59.2 and 59.1 points, respectively. However, employees showed higher variability (*SD* = 8.7) than students (*SD* = 4.7). The difference between the distributions [*M*_diff_ = 0.12 (−0.12, 0.37)] was associated with a negligible effect size [Hedges’ *g* = 0.02, (−0.01, 0.05)] and not statistically significant (*t*_6869.395_ = 0.99, *p* = 0.325)^4^. Hence, the aggregated data from 125 samples provide no robust evidence that students and employees would robustly differ in their levels IP expressions.

Finally, I addressed the question whether IP expressions have increased over time by computing a linear regression analysis predicting the CIPS scores by year of publication. The regression model showed that, on average, CIPS scores increased by *b* = 0.25 [(0.23, 0.26), *SE* = 0.01, *p* < 0.001] points per publication year. This translates to an effect size of β = 0.24. Further, the year of publication explained 5.7% of variance in CIPS scores. Hence, mean CIPS scores were higher in more recently published samples. However, as shown in Figure 1, this finding should be interpreted cautiously given the comparatively few data points before 2015 and the substantial increase in studies since. Thus, it is also reasonable that the precision of the population estimated increased over time, with higher imprecision in earlier years. The study of this trend requires further attention. The most appropriate approach to addressing this question would require tracking within-person change with longitudinal data over time to disentangle cohort effects from longitudinal trajectories reflecting changes in the IP over time. Also, the role of cohort effects, sample characteristics (e.g., sample composition, geographical and occupational differences), and other study-level characteristics should be addressed.

**Figure 1:**
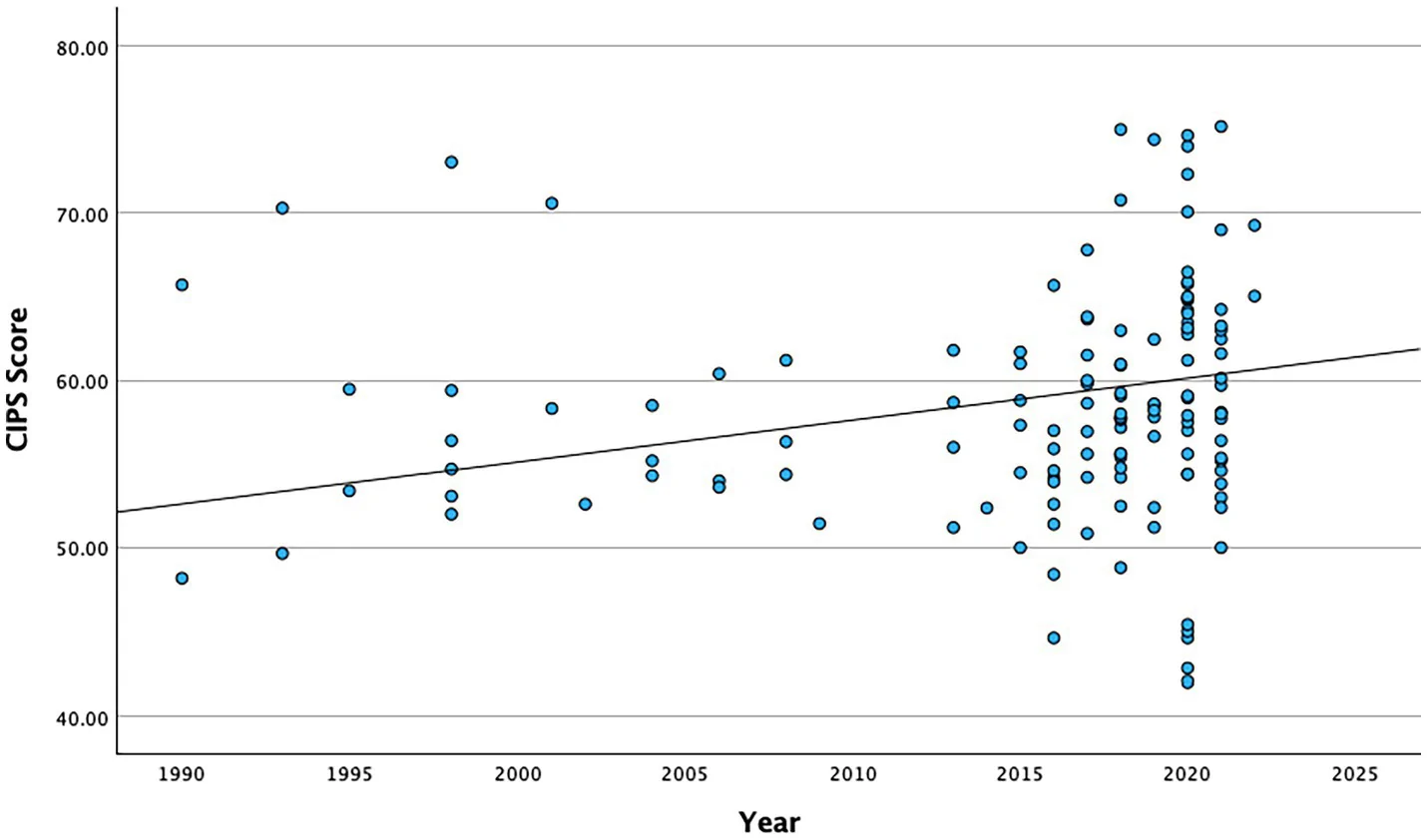
Distribution of CIPS scores in relation to studies’ year of publication.

Addressing these frequently discussed questions using a continuous approach with an illustrative data set derived from Stone-Sabali et al. (2023) literature review highlights that it is possible to use meta-analytic methods without relying on arbitrary and unvalidated cutoffs and “prevalence rates” that lack conceptual and empirical support in the context of the IP (Brauer and Proyer, 2026). The continuous approach provides an opportunity to clearly describe the degree of IP expressions as indicated by the mean and *SD* for samples and, when studying relationships with external variables (e.g., year of publication), to report unstandardized regression coefficients along with confidence intervals. This hopefully encourages future meta-analyses.

The initial findings support the notion that CIPS scores are approximately normally distributed on the sample level, with the means of the samples converging toward the mean of the normal distribution underlying the CIPS score continuum. Future research could extend these analyses and research questions in multiple ways. For example, a next step could involve coding the occupational and educational backgrounds of the samples and reanalyzing the distributions of the CIPS scores across, for example, medical students or STEM students. Interestingly, studies examining the IP in specific populations based on cutoffs often claim that these populations are particularly affected by the IP, as has been reported, for example, for dental students (Biloglav et al., 2026) and urologists (Jefferson et al., 2024). However, most studies report the expected rates of approximately 50 to 60% of participants being classified as showing high levels of IP according to a given cutoff. This aligns well with the assumption of a normal distribution, under which approximately 50% of scores would be expected to fall above the mean. Thus, relying on prevalence estimates based on arbitrary cutoffs without comparing study samples in terms of their sample means does not provide a well-supported basis for concluding that some populations show higher levels of IP expression than others. Hence, relying on means and measures of variability rather than prevalence estimates would allow for more thorough comparisons of study populations. This approach would also allow researchers to quantify differences in terms of raw scores and standardized effect sizes using a common metric, namely, the 20-to-100-point continuum of the CIPS.

## 7 Discussion

In extension to Brauer and Proyer’s (2026) earlier work illustrating the consequences of relying on unvalidated CIPS cutoffs, I discussed the lack of conceptual support for the categorical approach to the IP and illustrated the issues arising from the heterogeneity of estimates on basis of three recently published meta-analyses. Considering the conceptual, methodological, and empirical issues associated with relying on unvalidated and arbitrary cutoffs and conceptualizing the IP as a categorical construct, I suggest that the field align the analysis of the IP with its continuous conceptualization and move away from treating the IP as categorical (Brauer and Proyer, 2026; Clance and Lawry, 2024). To illustrate this approach, I provided an example meta-analysis addressing two frequently discussed questions: differences in IP expression between students and employees and changes in IP expression over time, which showed that these research questions can be addressed effectively using a continuous approach without relying on arbitrary cutoffs.

I hope that this work encourages future IP research to align its analytic methods with theoretical accounts and to learn more about the distribution of continuous scores within and across studies. Another fruitful avenue for future research is to extend the continuous approach from the CIPS total score to its recently identified facets, which capture fine-grained aspects of luck, discounting positive feedback, and fear of failure (Brauer and Proyer, 2025). Although studies have only recently begun to use this new framework, it may provide the field with an opportunity to learn more about how facet scores are distributed and how they relate to external variables across studies and populations.

## Data Availability

Publicly available datasets were analyzed in this study. This data can be found at: https://osf.io/7kp9f/.

## Footnotes

1 This perspective is also supported from Stone-Sabali et al.’s (2023) bibliographic review of the IP showing that *Personality and Individual Differences* is one of the major outlets for IP studies.

2 For further illustration, it might help to imagine such a erroneous description for other individual difference variables such as extraversion: The sentence “50% of the sample having extraversion” is equally meaningless as “50% of the sample having the IP.” Both traits are conceptualized as continuous dimensions.

3 The present manuscript focuses on the CIPS, as it is the most frequently used measure of the IP. To the best of my knowledge, alternative measures of the IP also do not provide validated cutoffs.

4 Since meta-analytic procedures that use sample size as weights for each observation, the degrees of freedom are also based on the weighting factor *n*. Accordingly, the *n* refers to the sample size of the cases that are used to weigh the means. Hence, the degrees of freedom for a two-independent-samples *t*-test are *n* + *n* − 2 = 13,383 + 5,521–2 = 18,902 (associated *t*-value = 1.25, *p* = 0.213). However, since the regular *t*-test assumes equal variances, a more realistic assumption relies on unequal variances of subgroups. To adjust the estimate, the Welch-Sattertwhaite correction is applied, with the test statistics reported in-text.
